# NOVEL STAIN SEPARATION METHOD FOR AUTOMATIC STEREOLOGY OF IMMUNOSTAINED TISSUE SECTIONS

**DOI:** 10.1093/geroni/igz038.958

**Published:** 2019-11-08

**Authors:** Palak Dave, Dmitry Goldgof, Lawrence O Hall, Saeed Alahmari, Peter R Mouton

**Affiliations:** 1 University of South Florida, Tampa, Florida, United States; 2 SRC Biosciences, Tampa, Florida, United States

## Abstract

Many studies of brain aging and neurodegenerative disorders such as Alzheimer’s and Parkinson’s diseases require rapid counts of high signal: noise (S:N) stained brain cells such as neurons and neuroglial (microglia cells) on tissue sections. To increase throughput efficiency of this work, we have combined deep learned (DL) neural networks and computerized stereology (DL-stereology) for automatic cell counts with low error (<10%) compared to time-intensive manual counts. To date, however, this approach has been limited to sections with a single high S:N immunostain for neurons (NeuN) or microglial cells (Iba-1). The present study expands this approach to protocols that combine immunostains with counterstains, e.g., cresyl violet (CV). In our method, a stain separation technique called Sparse Non-negative Matrix Factorization (SNMF) converts a dual-stained color image to a single gray image showing only the principal immunostain. Validation testing was done using semi- and automatic stereology-based counts of sections immunostained for neurons or microglia with CV counterstaining from the neocortex of a transgenic mouse model of tauopathy (Tg4510 mouse) and controls. Cell count results with principal stain gray images show an average error rate of 16.78% and 28.47% for the semi-automatic approach and 8.51% and 9.36% for the fully-automatic DL-stereology approach for neurons and microglia, respectively, as compared to manual cell counts (ground truth). This work indicates that stain separation by SNMF can support high throughput, fully automatic DL-stereology based counts of neurons and microglia on counterstained tissue sections.

